# Sjögren’s Syndrome Presenting With Multiorgan Extraglandular Manifestations: A Case Report

**DOI:** 10.7759/cureus.92628

**Published:** 2025-09-18

**Authors:** Gary Lail, Pratibha Thakur, Yaacov Bergman, Mariam Dabaghyan

**Affiliations:** 1 Internal Medicine, St. John's Episcopal Hospital, Far Rockaway, USA; 2 Internal Medicine, William Carey University College of Osteopathic Medicine, Hattiesburg, USA; 3 Internal Medicine, Touro College of Osteopathic Medicine, New York, USA

**Keywords:** case report, interstitial lung disease, myopericarditis, myositis, sjögren's syndrome, vasculitis

## Abstract

Sjögren’s syndrome (SS) is one of the most common autoimmune disorders. The disorder primarily affects the exocrine glands of the eyes and mouth and is regularly associated with extraglandular manifestations. These extraglandular manifestations can range from pulmonary, cardiac, muscular, neurological, to vascular. As the disease progresses, a majority of SS patients eventually develop extraglandular manifestations. Approximately 20% of patients with SS will develop pulmonary manifestations. However, it is uncommon to have the presenting symptoms be extraglandular and even less common to have multiple organs involved or critical illness. We present the case of a 42-year-old female whose only presenting symptoms were extraglandular, affecting multiple organs and leading to critical illness.

## Introduction

Sjögren's syndrome (SS) is an autoimmune disease process that is most commonly known for its exocrine gland manifestations. The lymphocytic invasion of exocrine glands, such as the salivary or lacrimal glands, characterizes these glandular manifestations [1]. SS has been associated with extraglandular symptoms involving pulmonary, cardiac, muscular, neurological, and vascular manifestations [1]. The pulmonary complications, such as interstitial lung disease (ILD), have been reported in about 20% of patients [2]. Vasculitis is also among the more common extraglandular presentations of SS [3]. SS associated with severe cardiac complications, such as myocarditis, is extremely rare, and documentation is mostly limited to case reports [4]. SS can also cause inflammatory myopathy accompanied by elevated creatine phosphokinase (CK) and abnormal electromyography, which occurs in only 1% of patients [5]. Among its less common outcomes, pulmonary embolism (PE) and deep vein thrombosis (DVT) are other rare and unexpected outcomes with an incidence rate of 3.9 and 2.8 per 1,000 person-years, respectively [6].

We report a rare case of a 42-year-old female with an atypical presentation of SS, notable for the multi-organ involvement, absence of sicca symptoms, and progression to critical illness requiring two hospitalizations.

## Case presentation

A 42-year-old female presented to the emergency room with a four-week history of a nonproductive cough. The patient reported mild shortness of breath and a persistent cough. History was notable for a two-day duration of bluish discoloration of the fingertips, and physical examination demonstrated persistent bluish discoloration. The patient’s vitals showed a fever of 100.9°F, but the remaining vitals were within the normal range. The patient was sent home with a five-day course of amoxicillin for possible pneumonia.

**Figure 1:**
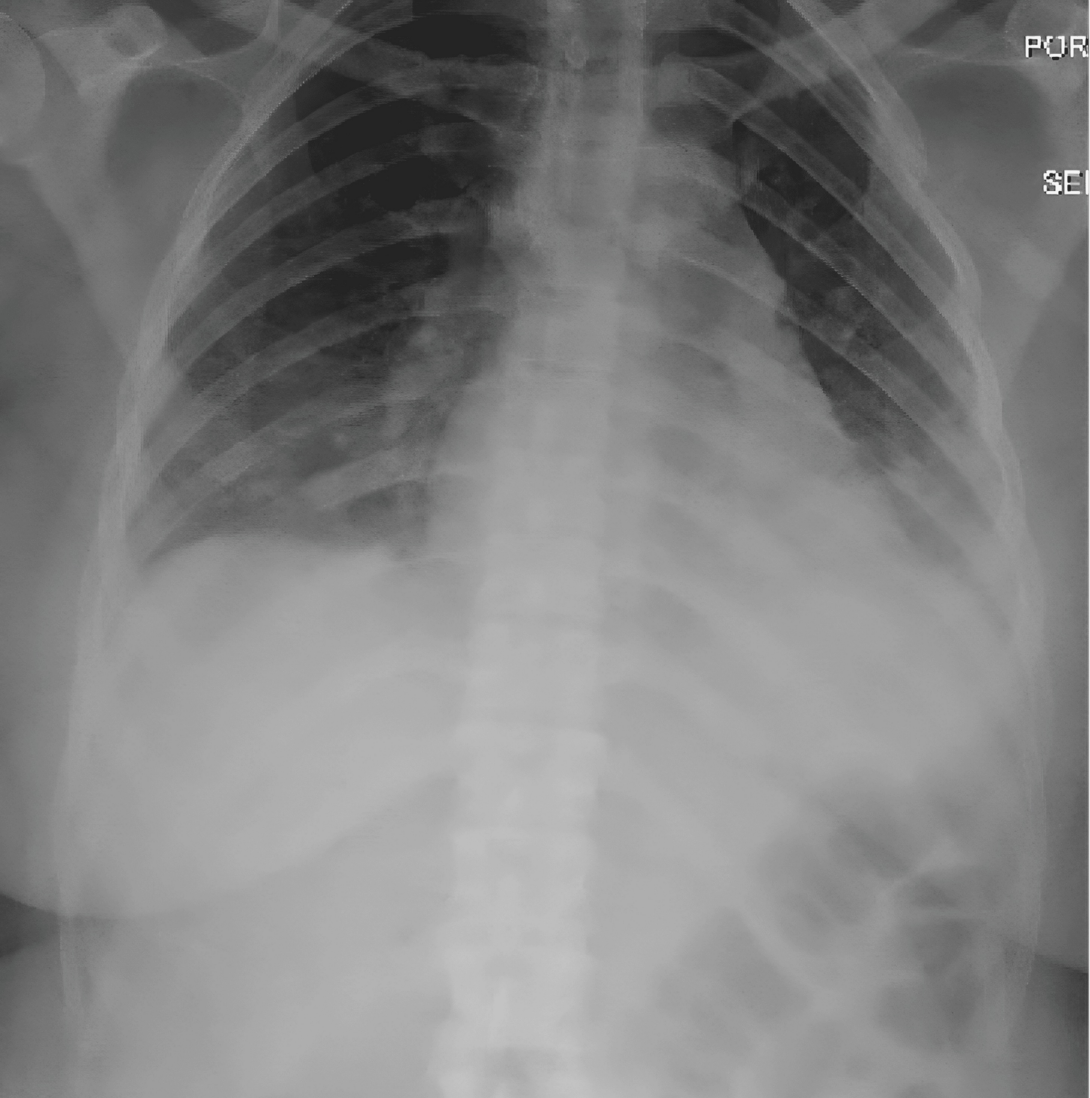
Portable chest X-ray: AP view. Persistent basilar opacities may be related to atelectasis or pneumonia. There is pulmonary vascular congestion, and no pleural effusion or cardiomegaly.

Seven days later, the patient returned to the emergency department due to a worsening cough and ongoing fevers. She continued to endorse a recurrent cough with shortness of breath. The physical exam revealed bluish fingertips with splinter hemorrhages in the nail bed. Review of systems was notable for myalgia, particularly involving the arms and legs. Laboratory evaluation revealed elevated creatine kinase (CK) levels. An X-ray of the patient’s chest displayed basilar consolidation (Figure 1). The patient was admitted to the floor due to treatment of sepsis from pneumonia, acute hypoxemic respiratory failure, and possible rhabdomyolysis. Infectious Disease was consulted, and she was given azithromycin 500 mg daily for five days. With no improvement in symptoms, she was switched to a two-day course of broad-spectrum antibiotics, vancomycin, and Zosyn IV at 12.5 mL/hr every eight hours for three days. The patient continued to have worsening swelling in her hands, fatigue, and no change in lung opacities or fever, along with an increase in CK, which led to a suspicion of possible sepsis from pneumonia vs infective endocarditis, and the patient was switched to meropenem.

**Figure 2:**
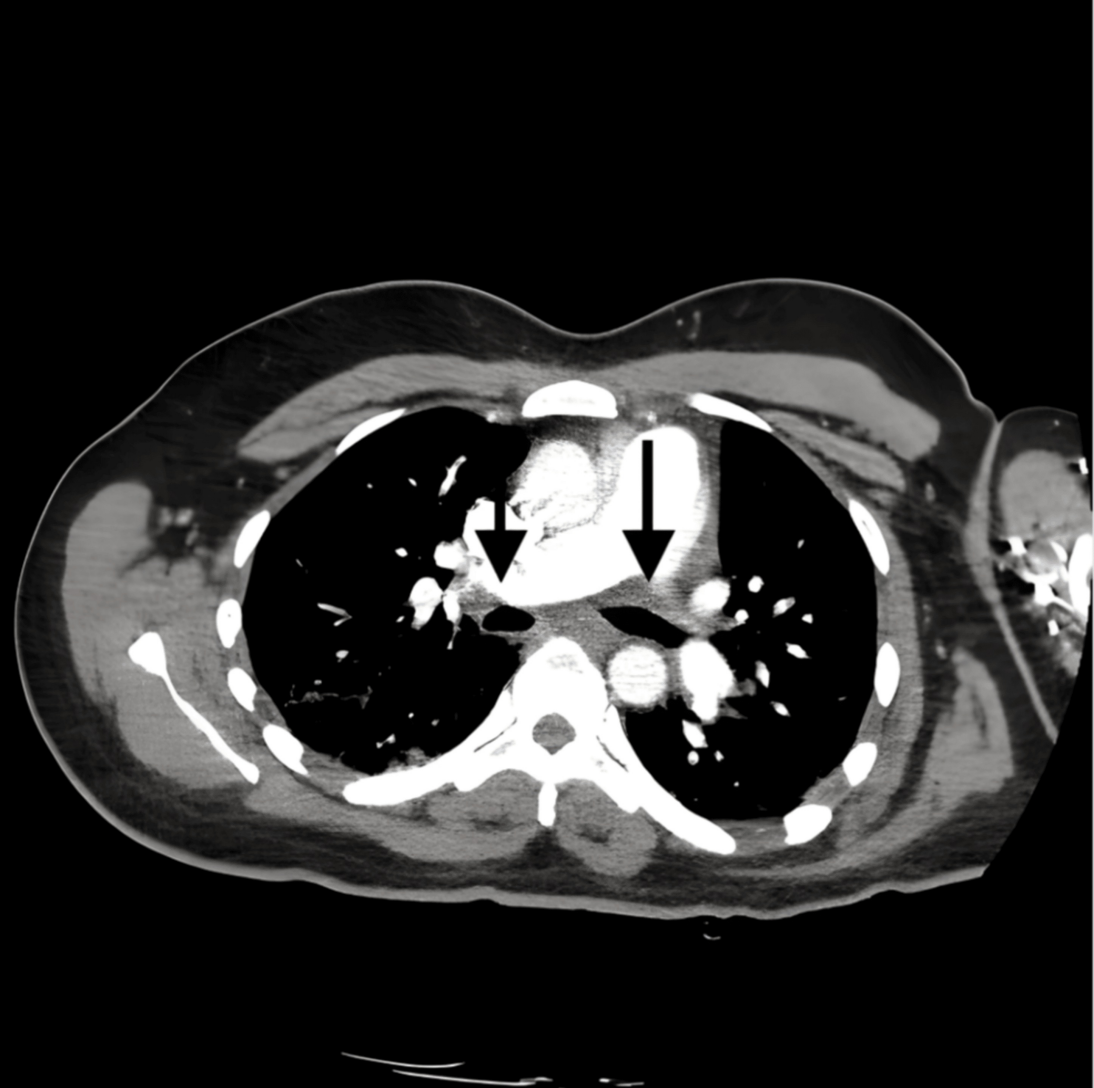
CT chest with intravenous contrast: axial view. This image demonstrates normal contrast opacification of the central pulmonary vasculature. Arrows: The pulmonary arteries are patent with no evidence of intraluminal filling defects, consistent with the absence of pulmonary embolism.

During the hospitalization, the patient was treated with broad-spectrum antibiotics for what was thought to be a primarily straightforward infectious etiology. Her labs were remarkable for negative Influenza A and B, RSV, and SARS-CoV-2. Her blood and urine cultures remained negative. The ongoing cough prompted a computed tomography (CT) chest (Figure 2), which showed irregular basilar-predominant consolidations, which were more noticeable on the left side. A transesophageal echocardiogram (TEEE) was obtained to evaluate the splinter hemorrhages and displayed no evidence of vegetations.

The patient continued to experience recurrent spiking fevers despite administration of broad-spectrum antibiotics and multiple negative infectious disease workups. The differential diagnosis was expanded to include autoimmune disorders. Serologic testing revealed elevated antinuclear antibody (ANA) levels and positive SSA/SSB antibodies, with increased RO52 and RO60, consistent with markers of SS. This was further supported by negative results for lupus anticoagulant, β2-glycoprotein antibodies, and anticardiolipin antibodies. In addition, rheumatoid factor (RF), cryoglobulins, scleroderma (Scl) antibodies, anti-centromere antibodies, and the extractable nuclear antigen (ENA) panel were all negative.

Her clinical progression and these additional findings were consistent with SS, with her presenting pulmonary symptoms considered non-glandular manifestations of the disorder. Treatment for SS was initiated. The patient was treated with a trial of an immunosuppressive medication regimen starting with methylprednisolone 60 mg, IV push for five days. Corticosteroid therapy was transitioned from IV to oral, with mycophenolate mofetil (Cellcept) 500 mg twice daily for two days until discharge, alongside prednisone 40 mg once daily. Within five days, her symptoms markedly improved. 

After five days of immunosuppressive therapy, the patient demonstrated marked improvement in pain and resolution of digital discoloration, along with a decline in CK levels from 4294 U/L to 2384 U/L, supporting the diagnosis of autoimmune-mediated myositis. Shortness of breath was significantly improved as well, confirming ILD due to a pulmonary manifestation of a rheumatological disease. The patient was discharged on mycophenolate 500 mg twice daily for immunosuppressive treatment. Trimethoprim-sulfamethoxazole 160/800 mg was given for *Pneumocystis jirovecii *pneumonia prophylaxis, and gabapentin 100 mg twice daily was prescribed for muscle pain.

Diagnostic assessment

During the first hospitalization, the ongoing cough and fever raised concerns for a possible infectious disease. However, all infectious workups, such as blood and urine cultures, were negative. The fingertip discolorations and splinter hemorrhages raised concern for possible endocarditis. Yet, a TEEE displayed no evidence of vegetations. The patient had a chest X-ray showing basilar consolidation (Figure 1). The ongoing cough prompted a follow-up CT chest (Figure 2), which showed irregular basilar-predominant consolidations, which were more noticeable on the left side. Due to the possible infectious nature of the symptoms, broad-spectrum antibiotics were administered throughout the hospitalization, yet there was no improvement in the ongoing symptoms. The ongoing symptoms, coupled with a lack of improvement following infectious disease protocols, raised suspicion for an underlying non-infectious process and led to an expanded hematologic workup.

During the second hospital admission, the initial CT imaging demonstrated a right middle-segmental artery PE. Ultrasound tests were not indicative of a DVT in the lower or upper extremities. However, elevated troponins raised concerns for a cardiac origin of the thrombosis, and subsequent MRI indicated possible Myocarditis.

Outcomes and follow-up

A month after her hospitalization, the patient presented to the emergency department with new fevers, cough, and shortness of breath despite adherence to prednisone/mycophenolate. The patient was visibly short of breath but had clear breath sounds. Furthermore, she was not hypoxic but was noted to have new extremity swelling. The patient denied chest pain but had an increased work of breathing, tachypneic, and tachycardic. EKG was non-actionable. Labs, viral panel, and computed tomography angiography (CTA) for PE evaluation was planned. CT chest showed a right middle-segmental artery pulmonary embolism. Ultrasound of the upper and lower extremities showed no deep venous thrombosis. Troponin was elevated at 856 ng/L. Cardiac MRI displayed subepicardial delayed enhancement in basal to mid inferior and inferoseptal walls, indicating possible myocarditis. Given the possibility of autoimmune myocarditis in the setting of worsening myopathy related to SS, the patient was discharged with metoprolol succinate 25 mg once daily, losartan 25 mg once daily, in addition to continuing her at-home medications, and a two-week cardiology follow-up and transthoracic echocardiogram in the next three months. The patient was also prescribed apixaban for anticoagulation. In addition, due to the sustained elevated CK and CK-MB, the mycophenolate was increased from 500 mg to 1000 mg twice daily in addition to prednisone 10 mg three times daily, with a recommended outpatient follow-up within the next month.

## Discussion

This case report discusses some of the serious extraglandular manifestations of the most common rheumatic disease, SS, in the form of an ILD, vasculitis, autoimmune myositis with myocarditis, and a PE. SS is an autoimmune disorder that most often presents with exocrine gland dysfunction, resulting in dry eyes or dry mouth in 98% of patients [7]. Careful attention to these symptoms is therefore usually highly suggestive of an underlying autoimmune process. In this case, however, the patient did not report sicca symptoms. Instead, her illness was marked solely by extraglandular involvement affecting multiple organ systems, leading to significant morbidity. This atypical presentation made establishing the final diagnosis considerably more difficult.

ILD is a serious and relatively common extraglandular manifestation of SS [2]. In this case, the absence of sicca symptoms likely contributed to the initial misdiagnosis of pneumonia. A higher degree of clinical suspicion for an underlying autoimmune disorder may have been warranted, as early recognition and treatment are critical to preventing irreversible lung damage. Vasculitis with splinter hemorrhages of the digits, as seen in this patient, represents another well-recognized extraglandular manifestation of SS [3]. In the absence of typical sicca symptoms, clinicians may be more inclined to attribute these findings to a more commonly associated condition, such as infective endocarditis, rather than an underlying autoimmune disease. Inflammatory myositis associated with SS is quite rare and can be difficult to detect if the patient has not been previously diagnosed with SS [5]. Furthermore, while SS can also affect other organ systems such as the kidneys, thyroid, and skin, cardiovascular involvement is rare and yet presents a significant burden for patients with primary SS [8]. In this patient, the myositis involved worsening inflammation extending from the skeletal muscles to the cardiac muscle, demonstrated by the elevated creatinine kinase, troponins, and MRI findings. 

In the absence of significant past medical history or known origins of emboli in this relatively young patient, the emergence of a pulmonary embolism (PE) was an unexpected finding. This PE could have been caused by a lower extremity DVT, given the calf pain, although the Doppler studies showed no evidence of it. There is also a clinical suspicion that the PE could have been due to an intracardiac thrombus developed because of the inflammatory myocarditis due to an underlying autoimmune disease [9]. Altered lipid metabolism due to exocrine dysfunction can lead to dyslipidemia in patients with SS [10], which can cause plaque formation and potentially a PE. Thus, it may be very possible that the underlying autoimmune flare may have played a significant role in the patient’s PE and critical condition. Even though this case was limited by the undetermined source of the PE, the lack of indications for a common cause suggests further investigation. Extraglandular manifestations, such as a hypercoagulable state due to chronic inflammation, vasculitis with endothelial dysfunction, and myositis, may suggest a Sjögren-associated manifestation of the PE.

## Conclusions

This case report emphasizes the complexity of disease presentation of an autoimmune disorder, SS, especially in cases involving clinical manifestations in vital organ systems such as the heart and lungs. The patient discussed in this case had a spectrum of rapidly developing symptoms at varying time points, ranging from common to rare, benign to severe, making it a very interesting presentation of SS. Although the patient did not exhibit the more commonly associated glandular symptoms of SS, the immunological markers pointed to SS. This highlights the importance of these immune markers with high specificity and sensitivity to SS in the diagnosis of SS, even in the absence of some commonly seen and expected clinical symptoms.

The importance of early disease recognition is imperative to initiating the appropriate treatment promptly due to early complications in the initial course of the disease. This is particularly important given the rapid progression to multiple organ dysfunctions within the first two months, as observed in this patient. Furthermore, an ED admission with new symptoms just one month after the initial discharge, despite ongoing immunosuppressive therapy, underscores the need to reassess medication management and follow-up. Thus, this case highlights the importance of early disease recognition and initiating appropriate treatment promptly due to early complications in the initial course of the disease.
